# Salivary Glucose Testing for Diabetes Mellitus: A Systematic Review and Meta-Analysis of Current Evidence and Methodological Heterogeneity

**DOI:** 10.3390/jcm15051829

**Published:** 2026-02-27

**Authors:** Kata Sára Haba, Patrik Krisztián Kreuter, Xinyi Qian, Gergely Agócs, Dorottya Bányai, Noémi Katinka Rózsa, Péter Hegyi, Péter Hermann, Carlos Jurado, Dóra Haluszka, Dániel Végh

**Affiliations:** 1 Centre for Translational Medicine, Semmelweis University, 1085 Budapest, Hungary; kreuter.patrik.krisztian@semmelweis.hu (P.K.K.); qian.xinyi@semmelweis.hu (X.Q.); agocs.gergely@semmelweis.hu (G.A.); banyai.dorottya@semmelweis.hu (D.B.); rozsa.noemi@semmelweis.hu (N.K.R.); hegyi.peter@semmelweis.hu (P.H.); hermann.peter@semmelweis.hu (P.H.); haluszkadora@gmail.com (D.H.); vegh.danel@semmelweis.hu (D.V.); 2 Department of Prosthodontics, Semmelweis University, 1085 Budapest, Hungary; 3 Department of Paediatric Dentistry and Orthodontics, Semmelweis University, 1085 Budapest, Hungary; 4 Department of Biophysics and Radiation Biology, Semmelweis University, 1085 Budapest, Hungary; 5 Institute of Pancreatic Diseases, Semmelweis University, 1085 Budapest, Hungary; 6 Institute for Translational Medicine, Medical School, University of Pécs, 7624 Pécs, Hungary; 7 Division of Operative Dent., Department of General Dentistry, College of Dentistry, The University of Tennessee Health Science Center, Memphis, TN 38104, USA; cjurado@uthsc.edu; 8 School of Dental Medicine, Ponce Health Sciences University, Ponce 00732, Puerto Rico

**Keywords:** diabetes mellitus, saliva, blood glucose, dentistry, oral medicine, meta-analysis

## Abstract

**Background/Objectives**: As current methods of measuring blood glucose levels are inconvenient and painful for patients, using salivary glucose as a non-invasive biomarker to estimate glucose levels may improve patient compliance. This study aims to quantify the association between salivary glucose levels and blood glucose levels and to assess how heterogeneity between studies and the methodological differences affect the potential clinical use of salivary glucose. **Methods**: The PRISMA guidelines were used for this review, and the protocol was registered on PROSPERO (CRD42023471213). Four databases were searched: PubMed, EMBASE, Cochrane’s Library Trials, and Web of Science. The search was conducted on 22 November 2023 and updated on 18 August 2025. No filters were applied for the search. Human studies, where paired salivary and blood samples were taken both from patients with diabetes and healthy individuals after at least 8 h of fasting, were included in the analysis. We extracted correlation coefficients and group means differences. Risk of bias was assessed with QUADAS-2, and the between-study heterogeneity was examined using random-effects models. **Results**: Through the systematic search, 15,162 articles were found, 25 of which were included in our meta-analysis. The analysis showed a weak correlation between whole-mouth saliva samples and blood samples (r^2^ value: 0.05) and a slightly stronger correlation between parotid saliva samples and blood samples (r^2^ value: 0.11). These low r^2^ values reflect weak associations and are reported descriptively. The difference between the mean salivary glucose level of patients with diabetes and controls was 4.43 mg/dL (95% CI: 2.05; 6.80). The high heterogeneity (I^2^ values approaching 100% for mean difference analyses) limits the interpretability of pooled estimates. **Conclusions**: Current evidence indicates that salivary and blood glucose levels associate weakly, and the study results are highly heterogeneous. Given the weak and highly heterogeneous associations observed across studies, current evidence does not support the use of salivary glucose measurement as an alternative to blood glucose measurement for the time being. Further standardized research is required before any conclusion about clinical applicability can be drawn.

## 1. Introduction

Diabetes mellitus (DM) is a chronic metabolic disorder affecting 540 million people worldwide, and this number is increasing; by 2045, it is expected to rise to 783 million [1]. In addition to confirmed cases, many patients remain undiagnosed [2]. The care of patients with DM places a heavy burden on society, and there is a need to improve their quality of life [3].

DM affects several organs, including the oral cavity, causing serious microvascular and macrovascular complications [4]. Patients with DM also have a higher rate of oral infections, such as fungal infections. Furthermore, DM also predisposes patients to periodontal disease and potentially deteriorates the status of pre-existing periodontitis [5]. Hyperglycemia not only elevates the frequency of infections and exacerbates hard tissue loss but also heightens the occurrence of xerostomia and hyposalivation, leading to burning mouth syndrome, diminished taste perception, and a greater prevalence of carious lesions [6].

Patients with DM need to regularly monitor their glucose levels to manage their condition effectively and reduce the risk of complications. Blood glucose measurement is a gold standard for controlling the metabolic status of patients with DM. However, it is inconvenient, causes pain, and is not cost-effective [7]. Two main types of blood glucose monitoring methods are known: point-of-care (POC) and continuous glucose monitoring (CGM). Although POC devices are generally effective, their accuracy can be affected by factors such as circulatory issues, e.g., dehydration or shock, as well as operator errors, e.g., inadequate sample volume or poor hygiene [8]. Repeated needle sticks can cause discomfort and deterioration in the quality of life of the patient [7]. CGM-based measurement systems, which operate subcutaneously or intravascularly, reduce the frequency of needle pricks and allow for continuous glucose monitoring. However, their accuracy can be affected by tissue perfusion and body temperature, and sensor application can be uncomfortable for patients [8]. A non-invasive, easy-access test would also significantly help diagnose patients with diabetes in countries where access to healthcare is severely limited. This is evidenced by the fact that most of our studies come from regions in India, where the number of people living with diabetes is around 89.8 million and rising [9]. On the other hand, the cost of health care and diagnostics in developed Western countries is expensive, so an easily available test, which could potentially be used as part of an annual dental check-up, could indicate if a patient’s glycemic control differs from the normoglycemic range.

These limitations have motivated interest in non-invasive approaches, including salivary glucose measurement, as a possible alternative for screening or monitoring. Previous studies have already shown that people with DM have changes in saliva composition in different salivary glands and have increased salivary glucose levels [7,10,11]. Glucose is not directly filtered out of the blood into saliva by passive diffusion but is facilitated by the SGLT1 (sodium-glucose cotransporter 1) protein. In addition to transporting glucose into the saliva, it also functions as a water channel, aiding water reabsorption. The expression of the SGLT1 protein is increased in diabetes mellitus, mainly in the parotid and submandibular glands [12]. These potential alterations in the amount of SGLT1 in the salivary glands raise the question of how salivary and blood glucose levels correlate. The already known alterations do not establish that salivary glucose reliably reflects the blood glucose level, and the true nature of this association remains uncertain [6].

The aim of this study was to compare the results of different clinical studies reporting paired fasting salivary and blood glucose measurements, and to quantify the strength and consistency of the association between the two values in both patients with and without DM. To ensure that saliva sample collection was not affected by DM-induced xerostomia and hyposalivation, patients were screened for these conditions in the included studies, and those affected were excluded. To prevent contamination by bacteria, digestive enzymes, and gingival cervical fluid, patients rinsed their mouths before saliva sample collection, then remained seated upright, permitting saliva to accumulate in the container until the requisite amount was attained, without monitoring the flow rate.

## 2. Materials and Methods

### 2.1. Search Strategy

This systematic review was based on the recommendations of the Cochrane Handbook, and the report was prepared according to the Preferred Reporting Items for Systematic Reviews and Meta-Analyses (PRISMA) statement items [13,14]; see Supplementary Materials S1 and S2. The protocol was developed and submitted to the International Prospective Register of Systematic Reviews (PROSPERO) database (ID: CRD42023471213) [14]. This study aimed to answer the following research question: How does the fasting salivary glucose level correlate with blood glucose level?

We adopted a PIRT-like organizational framework because all included studies reported paired fasting salivary and blood glucose measurements, and our synthesis procedures naturally required alignment across population, the two paired measurements, and their target relationship. Although the classical PIRT framework is often used in diagnostic accuracy studies, our aim was not to evaluate diagnostic performance but to quantify the statistical association between paired fasting salivary and blood glucose measurements. Therefore, we adapted PIRT into a non-diagnostic, measurement-comparison framework. The indicator and reference components refer to physiologically related measurements rather than index and reference tests in the diagnostic sense. This adaptation aligns the framework with the correlation-focused research question while preserving transparency in the conceptual organization of studies. The PIRT-inspired framework was used as follows:

- For population (P), we chose the general population.
- Indicator measurement (I) was a salivary glucose test based on the glucose-oxidase (GOD) method.
- Reference measurement (R), we chose the blood glucose test.
- The target relationship (T) was the correlation between salivary and blood glucose levels (r^2^ value). As a secondary outcome, we chose the group mean difference between diabetic and non-diabetic participants.

### 2.2. Data Sources and Search Strategy

Four databases were searched: PubMed, EMBASE, Cochrane’s Library Trials, and Web of Science. The search was conducted on 22 November 2023 and updated on the 18 August 2025. The core search string used across databases was:

("glucose" OR "sugar")

AND

("blood" OR "serum" OR "venous" OR "capillary" OR "glucometer" OR ("glucose" AND "kit") OR "glucose meter")

AND

("saliva" OR "salivary" OR "glucose oxidase" OR "glucose-oxidase")

Although the Boolean structure of the search terms was intentionally kept consistent across platforms for conceptual comparability, each query was executed using the database-specific interface, field structure, and syntax. No additional search filters were applied within any database. Full database-specific search strings and details are reported in Supplementary Material S3.

We prioritized a free-text-first strategy because several relevant studies, especially older ones, were not consistently indexed with MeSH or Emtree terms. PubMed’s automatic term mapping and Embase’s mapping implicitly incorporated these controlled vocabulary terms where available.

In addition, we intentionally kept the Boolean structure broad to avoid false-negative retrieval due to terminology heterogeneity. This approach ensured that potentially relevant studies were not lost at the search stage.

### 2.3. Eligibility Criteria

The following inclusion criteria were applied for this study: 1. Human clinical studies: randomized controlled trials, observational studies, and case series; 2. Patients fasted overnight before collecting both blood and salivary samples; 3. Patients rinsed their mouths before sample collection to avoid contamination with bacteria and cervical fluid; 4. Patients in the examined group were diagnosed with DM and confirmed by a clinician; 5. Patients were screened for xerostomia and hyposalivation and were excluded accordingly.

We did not restrict inclusion based on the specific assay brand or analytical platform, as they all use the glucose-oxidase method; only minor differences were reported.

Exclusion criteria were as follows: 1. Animal studies, case reports, review articles, 2. unavailable full-text version, 3. language other than English used for full-text version, 4. The fasting time needed to be increased, or the patients ate before sample taking.

Because our research question concerned the association between paired fasting salivary and blood glucose measurements, not the effect of an intervention or diagnostic accuracy performance, we included all primary human studies that provided such paired measurements, regardless of design (randomized trials, observational studies, cross-sectional studies, case–control studies, and case series). These designs can each contribute valid paired glucose data and therefore directly inform the association of interest. Differences in study design were expected to contribute to between-study variability; this was addressed by using random-effect models, design-agnostic extraction of paired biochemical measurements, and study-level risk-of-bias assessment. Because the outcome of interest is a biochemical measurement relationship, and not a clinical effect estimate, mixing study designs does not introduce the same conceptual limitations as in prognostic or interventional meta-analyses.

### 2.4. Study Selection and Data Extraction Strategy

First, two reviewers (K.S.H. and P.K.K.) independently screened titles and abstracts. Next, the same reviewers screened the full texts of all potentially eligible studies. Any disagreements were resolved through discussion and with the help of a third reviewer (X.Q.). After excluding studies that did not meet our eligibility criteria, we developed a standardized table for data extraction.

### 2.5. Quality Assessment

Two reviewers independently analyzed the articles for risk of bias using the QUADAS-2 tool [15]. The QUADAS-2 tool can assess the risk of bias as it contains four domains, each judged on a three-grade scale (low, unclear, high). Because our study objective was to quantify the association between paired fasting salivary and blood glucose measurements rather than diagnostic accuracy, we adapted QUADAS-2 by renaming “index” and “reference tests” to “indicator” and “reference measurements”, and tailored the signaling questions to emphasize near-simultaneous sampling after overnight fasting, standardized enzymatic saliva measuring procedures, and paired blood-saliva measurements. A summary of adapted signaling questions is provided in Supplementary Material S4. Any discrepancies between the two reviewers were resolved through discussion.

### 2.6. Statistical Analysis

Primary data collection and organization were carried out using Microsoft Excel. Statistical analysis was performed using R version 4.1.3.

Where graphically available, individual patient data (IPD) for blood and salivary glucose levels were extracted using WebPlotDigitizer v4.7 [16]. This method is inherently approximate, as it depends on axis scaling, figure resolution, and potential point overlaps; therefore, extracted values should be considered estimates rather than exact reproductions of the original data.

For each study with IPD, we fitted a simple linear regression model using the lm() function of the {stats} package with salivary glucose level as the response variable and blood glucose level as the explanatory variable, and we calculated the coefficient of determination (r^2^) to describe the proportion of variance in salivary glucose explained by blood glucose. Transforming correlation coefficients using Fisher’s z and conducting a formal pooled-correlation meta-analysis were not feasible, as many studies did not provide sufficient information (e.g., standard errors or confidence intervals) and used heterogeneous analytical approaches, which limited the feasibility of statistically coherent pooling. Due to this heterogeneity, the r^2^ values are reported solely as descriptive, study-level indicators and are not used for inferential or clinical interpretation. To explore a potential overall linear relationship, we attempted to fit a linear mixed-effects model to the combined IPD (salivary glucose level as response, blood glucose level as explanatory variable, random intercept for study) using the lmer() function of the {lme4} package [17] version 1.1.31; however, substantial between-study variability and model diagnostics indicated that assumptions were not met, so this global model was not used for inferential purposes. Fitting a global model was also attempted for a subset of IPD, specifically based on parotid salivary samples, which yielded similarly poor-fitting and model diagnostic results. The analysis of IPD outcomes is summarized in scatterplots. Each study is indicated by a separate color represents each study. Background shades represent ranges of normoglycemic (65–110 mg/dL; green), impaired glucose tolerance (IGT; 110–125 mg/dL; orange), and diabetic (>125 mg/dL; red) fasting blood glucose levels.

We used the metacont() function of the [18] package (version 6.1.0 [19]) to compare the summary statistics of non-euglycemic and normoglycemic patients; the effect size was the mean difference (MD) in blood as well as salivary glucose levels. We anticipated considerable between-study heterogeneity, so we used a random-effects model to pool effect sizes. Given the expected heterogeneity, pooled mean differences were reported for completeness but prespecified to be interpreted descriptively if heterogeneity exceeded conventional thresholds. To calculate the between-study variance (tau squared), we used the restricted maximum likelihood estimator [20]. We used Knapp-Hartung adjustments [21] to calculate the confidence interval around the pooled effect. We used funnel plots and Egger’s regression test for funnel plot asymmetry to assess small study effects. We identified studies with strong influence on our overall results using the InfluenceAnalysis() function of the {dmetar} package [22].

Finally, we attempted to pool the summary statistics (means) of salivary glucose levels (response variable) and blood glucose levels (explanatory variable) from all included studies using linear meta-regression. First, we fitted a linear mixed-effects meta-regression model to the data of non-euglycemic as well as normoglycemic patients, separately, using the rma() function of the {metafor} (version 3.8.1 [23]). Then, we fitted all data with a multivariate/multilevel linear mixed-effects meta-regression model using the rma.mv() function of the {metafor} package; the random intercept variables were diabetes status and study. Again, the modeling yielded a very poor fit and model diagnostics revealed that modeling conditions were not met. Again, scatterplots were used to summarize the analysis results, with similar settings as for the IPD analysis.

### 2.7. Sensitivity Analysis

To evaluate whether digitization error could have influenced our conclusions, we conducted a plausibility sensitivity analysis based on hypothetical perturbations of ±5% and ±10% of the extracted salivary and blood glucose values. This range reflects a conservative estimate, well above the typical uncertainty reported for WebPlotDigitizer when axis calibration is adequate [24,25].

Small proportional perturbations in digitized values affect both covariance and variance by similar factors; therefore, the resulting r^2^ values shift only minimally because they depend on the squared ratio of these quantities. For the individual-study correlation models, r^2^ values were 0.05 (whole-mouth saliva) and 0.11 (parotid saliva). Even under a hypothetical ±10% perturbation of all digitized values, the resulting r^2^ values would vary only minimally (approximately ±0.01–0.03), remaining well within the range interpreted as weak associations. Therefore, digitization error cannot plausibly elevate these values to a level indicating moderate or strong correlation.

For the meta-analysis of mean differences, the observed difference in salivary glucose levels between hyperglycemic and normoglycemic participants was 4.43 mg/dL (95 CI: 2.05; 680). Perturbing means and standard deviations by ±5% or ±10% changes the magnitude of the difference only slightly (approximately ±1–2 mg/dL) and does not alter the direction or the statistical significance of the effect.

Taken together, this sensitivity analysis demonstrates that plausible levels of digitization error would not materially influence the conclusions of our review. The weak association and high heterogeneity reported are therefore unlikely to be attributable to digitization artifacts.

## 3. Results

### 3.1. Selection Process

**Figure 1:**
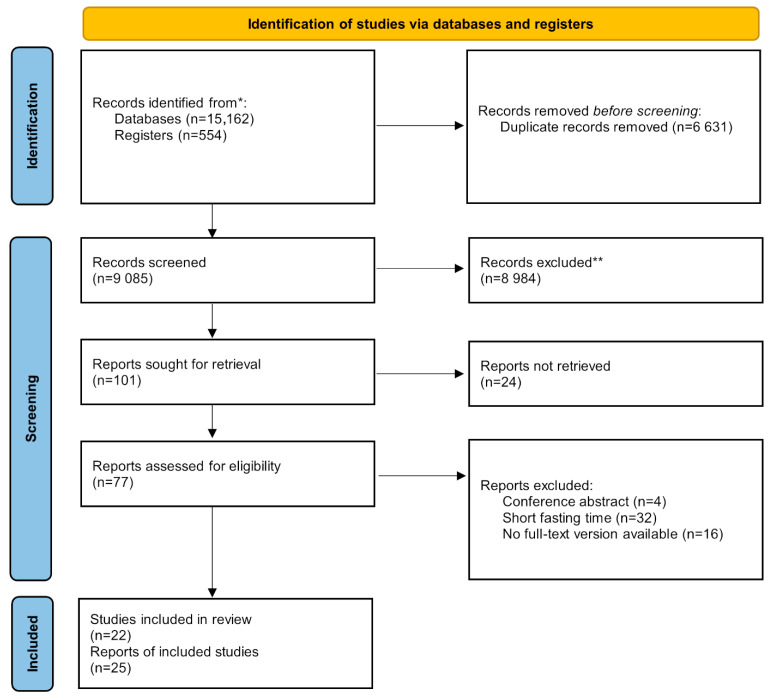
PRISMA 2020 Flowchart (* See Supplementary Material S3 for further details on the database search queries used. ** See Supplementary Material S5 for further details on the exclusion criteria.).

The systematic search identified a total number of 15,716 records across databases and registers: PubMed (n = 4175), EMBASE (n = 6710), Cochrane’s Library Trials (n = 554), and Web of Science (n = 4277). After duplicate removal, 9085 studies were left. After selection based on title and abstract, 101 studies remained for full-text selection. See Supplementary Material S5 for more details on the excluded articles. At the end of the selection process, we included 25 studies that met our objectives using the adapted PIRT framework, with a particular focus on fasting time, specifically overnight fasting, for which full-text versions were available (see Figure 1).

### 3.2. Characteristics of Studies Included

The following types of studies were identified: observational studies (n = 22), a randomized controlled trial (n = 1), a case–control (n = 1), and a cross-sectional study (n = 1). We included all study designs, as they can all provide information on the underlying association between salivary and blood glucose levels; however, they differ in their susceptibility to selection bias, confounding factors, and measurement bias, which were expected to contribute to between-study heterogeneity. This was taken into consideration by using random-effects models. In all cases, the index test was the salivary glucose test, and the reference test was the blood glucose test. The particular test types for each study are detailed in Supplementary Material S6. The population was a general population of patients with DM, patients with impaired fasting glucose, and healthy people of all ages and sexes, as shown in Table 1.

### 3.3. Results of Individual Patient Data Analysis for Whole-Mouth Saliva

**Figure 2:**
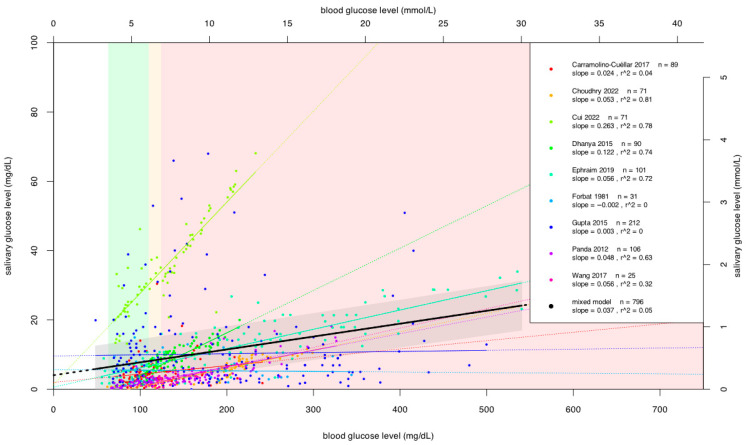
Regression models of whole-mouth salivary glucose level as a function of blood glucose level based on individual patient data. Units of measurement are both in mmol/L and mg/dL. Colored ranges (based on blood glucose levels): green: normoglycemic; yellow: impaired fasting glucose; red: DM. Each dot represents an individual patient, thin lines represent regression functions for each study, the thick black line represents the mixed-effect linear regression model fit, and the gray area represents its confidence interval band [7,10,11,26,28,29,30,31,35].

Individual patient data were extracted from nine studies [7,10,11,26,28,29,30,31,35] using the WebPlotDigitizer software, and data on blood and salivary glucose levels were determined for a total of 796 patients. We fitted a linear regression model to the data from each study, as well as a linear mixed-effects model to the entire data set. Individual data and fitted lines are shown in a scatterplot (see Figure 2). The coefficient of determination (r^2^) for the mixed-effects model was 0.05, indicating a poor fit. This value reflects only the proportion of variance explained in this descriptive model and is not intended as an inferential measure of predictive or clinical performance. For a better insight, a meta-regression analysis was also carried out; see Supplementary Material S7 for details.

### 3.4. Results of Analysis of Individual Patient Data on Parotid Saliva

**Figure 3:**
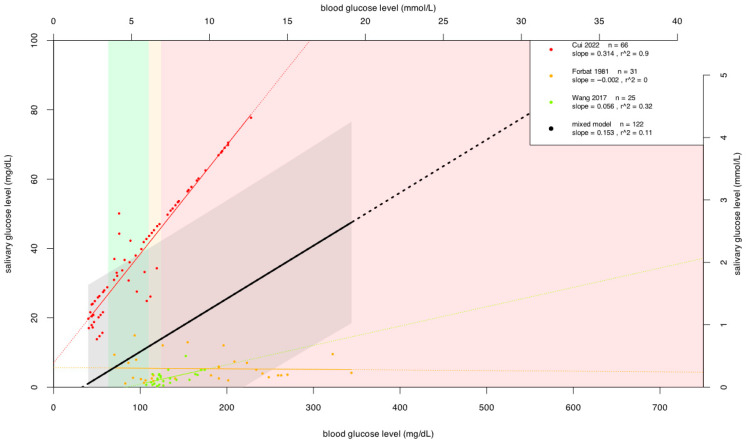
Individual patient data on parotid saliva. Units of measurement: mmol/L and mg/dL both. The green range represents normoglycemic patients, the yellow range represents patients with impaired fasting glucose, and the red range represents DM for blood glucose level. Each dot represents an individual patient data point, the colored thin lines represent the correlation lines for each article, and the thick black line represents the mixed model fit [10,11,26].

On the basis of literature recommendations, we investigated the correlation between saliva isolated from the parotid gland and blood glucose levels. After analyzing IPD from three articles [10,11,26], we found that the r^2^ value was 0.11, as shown in Figure 3. Similarly, the r^2^ value should be interpreted descriptively, given the methodological heterogeneity of the included studies.

### 3.5. Forest Plots from Mean Differences Between Salivary and Blood Glucose Levels

**Figure 4:**
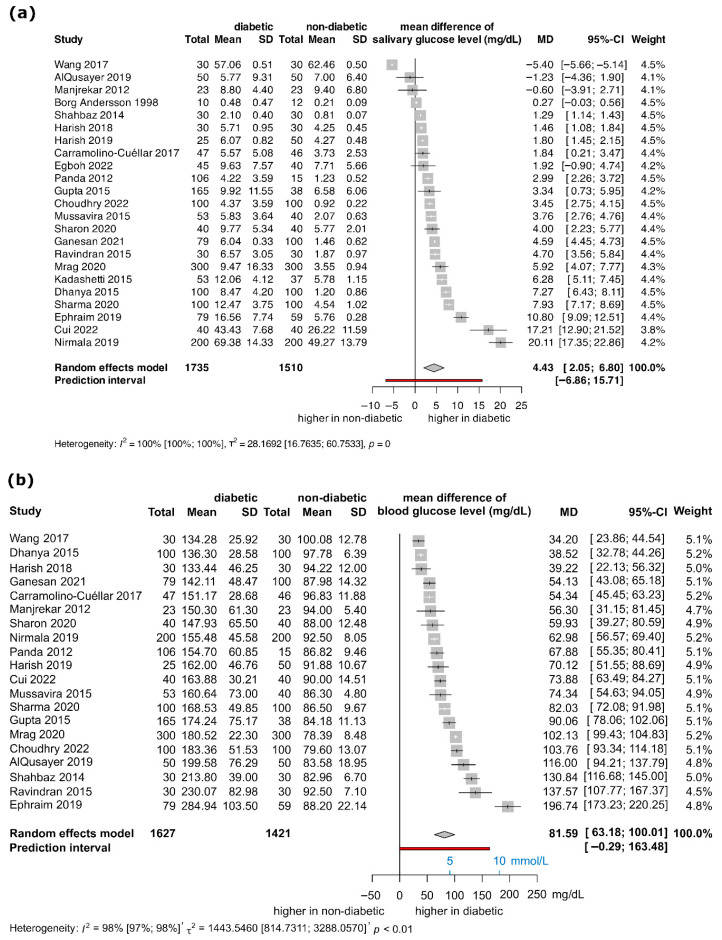
Forest plot showing the meta-analysis of the mean differences (mean of the DM group minus mean of non-DM group) between salivary glucose levels (**a**) and blood glucose levels (**b**) [7,11,26,28,29,30,31,32,33,34,35,36,37,38,39,40,41,42,43,44,45,46,47].

The forest plots in Figure 4 show the distribution of mean saliva and blood glucose levels extracted from the articles at 95% confidence intervals. The results indicate statistically significant (*p* < 0.05) between-group differences on both Figure 4a,b, however the high heterogeneity and small magnitude (I^2^ = 100%, *τ*^2^ = 28.1692; and I^2^ = 98%, *τ*^2^ = 1443.5460 for plots a and b, respectively) limit their clinical relevance, and the pooled estimate should be interpreted as explanatory rather than inferential.

### 3.6. Risk of Bias Analysis with QUADAS-2 Tool

The QUADAS-2 risk of bias tool [15] was used to evaluate the quality of methodologies used in the articles included. The risk levels for the selected articles in the four domains are detailed in Table 2, based on the investigators’ decisions. Note that the signaling questions were adapted for an association-focused, paired measurement question (see Supplementary Material S4).

## 4. Discussion

This systematic review and meta-analysis was performed to investigate the relationship between salivary and blood glucose levels in patients with DM and healthy individuals. Our analytical strategy focused mainly on descriptive IPD regression and meta-analyses of mean differences rather than on conventional pooled-correlation meta-analyses. We chose to present study-specific correlation measures and IPD-based models descriptively, while using random-effects models to summarize between-group contrasts. The results of the meta-analysis show that although there is a difference in salivary glucose levels between the non-euglycemic and healthy groups (significant MD observed), these group-level contrasts do not imply adequate diagnostic performance at the individual level, and the pooled mean difference is not clinically interpretable due to the very high heterogeneity, serving only as a descriptive indicator of direction rather than a meaningful summary effect.

As mentioned in the introduction, glucose transport into saliva does not occur by simple diffusion, but by facilitated transport with SGLT1 proteins. However, the number of receptors required for this may increase with higher blood glucose levels as gene expression of the receptors increases. Higher receptor numbers may result in higher concentrations of glucose in saliva [12].

First, we investigated the relationship between total oral non-stimulated saliva samples of diabetic and control patients and their blood glucose levels after overnight fasting. Scatter plots suggested a likely, but not significant correlation between blood glucose and salivary glucose levels. We also observed high heterogeneity, which can likely be explained by the inclusion of diverse study designs (RCTs, observational studies, case–control studies and case series) with a wide range of methodological and analytical methods.

The difference between the results of each study could also be explained by different storage conditions after sampling: in 12 cases (Gupta et al., Cui et al., Wang et al., Choudhry et al., Borg-Andersson et al., Ganesan et al., Harish et al. 2018, Harish et al. 2019, Mussavira et al., Nirmala et al., Ravindran et al., Shahbaz et al. [7,11,26,29,34,36,37,38,42,43,44]), samples were stored refrigerated, and in 2 cases, even sampling tubes were pre-chilled (Cui et al., Mussavira et al. [11,42]). In the other 11 cases (Panda et al., Dhanya et al., Ephraim et al., AlQuyaser et al., Egboh et al., Carramolino-Cuéllar et al., Kadashetti et al., Manjrekar et al., Mrag et al., Sharma et al., Sharon et al. [28,30,31,32,33,35,39,40,41,46,47]), there is no information on whether samples were stored in cold storage. The study by Forbat et al. [10] reported that the cooling temperature was below 4 degrees Celsius.

Saliva volume used for sample collection was not uniform either: in most cases, no specific amount was mentioned, but where it was, the lowest value was 1.5 mL (Borg-Andersson et al., Ravindran et al. [34,44]), 2 mL was mentioned in the studies by Sharma et al., Egboh et al., and Gupta et al. [7,33,46], 3 mL in the study by Ganesan et al. [36], and a maximum of 10 mL was mentioned in the study by AlQuyaser et al. [32].

Another important physiological difference between blood and saliva is that the latter has a higher proportion of various sugar-degrading enzymes, such as amylase [12]. Although amylase is mainly involved in the breakdown of starch, bacteria in the oral cavity enzymatically break down the glucose thus released. The carbohydrate release of glycoproteins, also present in higher concentrations in saliva, may also affect measurement results. Glucose consumption by bacteria in the oral cavity may also affect salivary glucose levels [48]. Avoiding contamination would be very difficult, and the measurement would no longer necessarily remain non-invasive. Therefore, after the first analysis, the literature suggested analyzing the correlation between blood glucose levels and glucose levels in saliva samples from the parotid gland only [7]. These comparisons gave better results in some cases, but parotid collection is more technically demanding and time-consuming than unstimulated whole mouth sample taking. As in the first analysis, data were collected for individual patients. Here, the r^2^ value showed that the correlation was stronger; however, it still remained moderate to support clinical applicability, and even if it offered better analytical performance, its current collection requirements would restrict its translational applicability in everyday clinical practice.

Other factors, such as salivary flow rate, alcohol consumption, or even diet type, could also potentially distort analysis results [48]; however, these were not discussed in the articles included. As mixed-model analyses showed a weak relationship between salivary and blood glucose levels when all studies were included, we also carried out subset analyses based on blood glucose levels. Where only healthy patients were included, the results were statistically significant; this reinforces the assumption that altered physiological conditions in patients with diabetes result in substantial changes in salivary glucose levels.

These factors (salivary contamination, variable flow rate, recent diet and alcohol intake, and inconsistent storage conditions) can all alter salivary glucose independently of blood glucose and thus bias the observed association. Because they were reported and handled inconsistently across studies, they likely contribute to the substantial between-study heterogeneity and reduce confidence in translating salivary glucose measurements into clinical practice.

### 4.1. Strengths and Limitations

The strength of our study lies in its highlighting of the importance of a non-invasive testing method, which warrants further investigation. However, there are limitations, including the need for more studies and longer follow-ups.

IPDs were reconstructed from published scatterplots using the WebPlotDigitizer tool, which may introduce minor measurement error. However, as our sensitivity analysis shows, these inaccuracies are very unlikely to affect our findings based on the observed weak association found in the studies.

Additionally, we only included full-text studies published in English; this restriction may have introduced language bias and reduced the completeness and global generalizability of our findings and should be considered when interpreting the results.

While our methodological approach may reduce statistical efficiency, it also avoids potentially misleading pooled correlation estimates under incompatible assumptions, and it is consistent with our main conclusion, that the association between salivary and blood glucose is weak and highly heterogeneous across studies, as reflected by small, descriptive r^2^ values that were not used for inference.

### 4.2. Implications for Practice and Research

The translation of scientific results into clinical practice is crucial [49,50]. Because this review quantified statistical association rather than agreement or diagnostic accuracy, the findings should not be interpreted as evidence for or against clinical interchangeability. The consistently weak associations and substantial methodological heterogeneity instead indicate that salivary glucose cannot currently be evaluated for clinical substitution, and further standardized methodological work is needed to enable such assessments.

## 5. Conclusions

The findings reveal poor correlation coefficients, indicating only a weak association between salivary and blood glucose levels. Furthermore, high between-study heterogeneity limits confidence in the pooled estimates. The significant variability in methodologies, such as sample storage conditions and collection techniques, undermines the reliability of the studies. While we did not evaluate accuracy indices regarding salivary glucose, such as specificity, sensitivity or ROC curves, our findings indicate that the current evidence is insufficient to support considering salivary glucose as an alternative for blood glucose monitoring. Therefore, blood glucose level measurement remains the “gold standard”, while salivary glucose testing remains a possible alternative that needs further research to support its eventual translation from fundamental research into clinical practice.

## Figures and Tables

**Table 1:** List of the included studies (n = 25)—Egboh, Harish, Kadashetti and Sharma studies contained two or more study groups.

First Author	Year	Number of Participants	Study Type	Exposure	Control
Gupta [7]	2015	165	observational	T2DM	healthy adults
Cui [11]	2022	40	observational	DM	healthy adults
Wang [26]	2017	30	observational	T2DM	healthy adults
Sreedevi [27]	2008	60	observational	DM	healthy adults
Panda [28]	2012	106	observational	T2DM	healthy adults
Forbat [10]	1981	31	observational	DM	N/A
Choudhry [29]	2022	100	observational	DM	healthy adults
Dhanya [30]	2016	100	observational	DM	healthy adults
Ephraim [31]	2019	79	case–control	DM	healthy adults
AlQusayer [32]	2019	50	cross-sectional	DM	healthy adults
Egboh [33]	2022	23	RCT	T2DM male	healthy adult males
		22	RCT	T2DM female	healthy adult females
		56	RCT	T2DM	healthy adults
Borg [34]	1998	10	observational	T2DM	healthy adults
		10	observational	IGT	healthy adults
Carramolino-Cuéllar [35]	2017	47	observational	T2DM	healthy adults
Ganesan [36]	2022	79	observational	TIDM	healthy adults
Harish [37]	2018	30	observational	T2DM	healthy adults
Harish [38]	2019	25	observational	T2DM uncontrolled	healthy adults
		25	observational	T2DM controlled	healthy adults
Kadashetti [39]	2015	21	observational	T2DM 0 > 200 mg/dL)	healthy adults
		32	observational	T2DM (130–200 mg/dL)	healthy adults
		53	observational	T2DM	healthy adults
Manjrekar [40]	2012	23	observational	T2DM	healthy adults
Mrag [41]	2020	300	observational	T2DM	healthy adults
Mussavira [42]	2015	53	observational	DM	healthy adults
Nirmala [43]	2019	200	observational	DM	healthy adults
Ravindran [44]	2015	30	observational	T2DM	healthy adults
Shahbaz [45]	2014	30	observational	TIDM	healthy children
Sharma [46]	2020	100	observational	DM, venal	healthy adults
		100	observational	DM, capillary	healthy adults
Sharon [47]	2020	40	observational	T2DM	healthy adults

**Table 2:** Risk of bias analysis.

Study	Patient Selection	Indicator Measurement (Salivary)	Reference Measurement (Blood)	Flow and Timing	Overall
AlQusayer (2019) [32]	Unclear	Low	Low	Low	Unclear
Borg (1998) [34]—T2DM	Low	Low	Low	Low	Low
Borg (1998) [34]—IGT	Low	Low	Low	Low	Low
Carramolino-Cullar (2017) [35]	Low	Low	Low	Unclear	Unclear
Choudhry (2022) [29]	Low	Low	Low	Low	Low
Cui (2022) [11]	Low	Low	Low	Low	Low
Dhanya (2016) [30]	Low	Low	Low	Low	Low
Egboh (2022) [33]—Combined	Low	Low	Low	Low	Low
Egboh (2022) [33]—Female	Low	Low	Low	Low	Low
Egboh (2022) [33]—Male	Low	Low	Low	Low	Low
Ephraim (2019) [31]	Low	Low	Low	Low	Low
Forbat (1981) [10]	High	Unclear	Low	High	High
Ganesan (2022) [36]	Low	Low	Low	Low	Low
Gupta (2015) [7]	Low	Low	Low	Low	Low
Harish (2018) [37]	Low	Low	Low	Low	Low
Harish (2019) [38]—Controlled	Low	Low	Low	Low	Low
Harish (2019) [38]—Uncontrolled	Low	Low	Low	Low	Low
Kadashetti (2015) [39]—BG > 200 mg/dL	Low	Low	Low	Low	Low
Kadashetti (2015) [39]—BG: 130–200 mg/dL	Low	Low	Low	Low	Low
Kadashetti (2015) [39]—Overall	Low	Low	Low	Low	Low
Manjrekar (2012) [40]	Unclear	Low	Low	Low	Unclear
Mrag (2020) [41]	Low	Low	Low	Low	Low
Mussavira (2015) [42]	Low	Low	Low	Low	Low
Nirmala (2019) [43]	Low	Low	Unclear	Low	Unclear
Panda (2012) [28]	Low	Low	Low	Low	Low
Ravindran (2015) [44]	Low	Low	Low	Low	Low
Shahbaz (2014) [45]	Low	Low	Low	Low	Low
Sharma (2020) [46]	Low	Low	Unclear	Low	Unclear
Sharon (2020) [47]	Low	Low	Low	Low	Low
Sreedevi (2008) [27]	Unclear	Low	Low	Low	Unclear
Wang (2017) [26]	Low	Low	Low	Low	Low

## Data Availability

The raw data supporting the conclusions of this article will be made available by the authors on request.

## Supplementary Materials

The following supporting information can be downloaded at: https://www.mdpi.com/article/10.3390/jcm15051829/s1. S1. PRISMA 2020 Main Checklist; S2. PRISMA 2020 Abstract Checklist; S3. Search keys and number of articles retrieved; S4. Adapted QUADAS-2 signaling questions for paired salivary-blood glucose studies; S5. Excluded articles; S6. Data on storing conditions, analysis methods, and volume of salivary samples; S7. Meta-regression for whole-mouth saliva and blood glucose samples.
