# Supplementary material for: Salivary Glucose Testing for Diabetes Mellitus: A Systematic Review and Meta-Analysis of Current Evidence and Methodological Heterogeneity
Source: J Clin Med. 2026 Feb 27;15(5):1829. doi: 10.3390/jcm15051829 (PMC12986110; doi:10.3390/jcm15051829)

Supplementary materials

S1. PRISMA 2020 Main Checklist

Topic	No.	Item	Location where item is reported
**TITLE**			
**Title**	1	Identify the report as a systematic review.	1
**ABSTRACT**			
**Abstract**	2	See the PRISMA 2020 for Abstracts checklist	
**INTRODUCTION**			
**Rationale**	3	Describe the rationale for the review in the context of existing knowledge.	3
**Objectives**	4	Provide an explicit statement of the objective(s) or question(s) the review addresses.	4
**METHODS**			
**Eligibility criteria**	5	Specify the inclusion and exclusion criteria for the review and how studies were grouped for the syntheses.	5
**Information sources**	6	Specify all databases, registers, websites, organisations, reference lists and other sources searched or consulted to identify studies. Specify the date when each source was last searched or consulted.	5
**Search strategy**	7	Present the full search strategies for all databases, registers and websites, including any filters and limits used.	5
**Selection process**	8	Specify the methods used to decide whether a study met the inclusion criteria of the review, including how many reviewers screened each record and each report retrieved, whether they worked independently, and if applicable, details of automation tools used in the process.	6
**Data collection process**	9	Specify the methods used to collect data from reports, including how many reviewers collected data from each report, whether they worked independently, any processes for obtaining or confirming data from study investigators, and if applicable, details of automation tools used in the process.	5
**Data items**	10a	List and define all outcomes for which data were sought. Specify whether all results that were compatible with each outcome domain in each study were sought (e.g. for all measures, time points, analyses), and if not, the methods used to decide which results to collect.	5
	10b	List and define all other variables for which data were sought (e.g. participant and intervention characteristics, funding sources). Describe any assumptions made about any missing or unclear information.	5
**Study risk of bias assessment**	11	Specify the methods used to assess risk of bias in the included studies, including details of the tool(s) used, how many reviewers assessed each study and whether they worked independently, and if applicable, details of automation tools used in the process.	6
**Effect measures**	12	Specify for each outcome the effect measure(s) (e.g. risk ratio, mean difference) used in the synthesis or presentation of results.	6
**Synthesis methods**	13a	Describe the processes used to decide which studies were eligible for each synthesis (e.g. tabulating the study intervention characteristics and comparing against the planned groups for each synthesis (item 5)).	6
	13b	Describe any methods required to prepare the data for presentation or synthesis, such as handling of missing summary statistics, or data conversions.	6-7
	13c	Describe any methods used to tabulate or visually display results of individual studies and syntheses.	6-7
	13d	Describe any methods used to synthesize results and provide a rationale for the choice(s). If meta-analysis was performed, describe the model(s), method(s) to identify the presence and extent of statistical heterogeneity, and software package(s) used.	6-7
	13e	Describe any methods used to explore possible causes of heterogeneity among study results (e.g. subgroup analysis, meta-regression).	6-7
	13f	Describe any sensitivity analyses conducted to assess robustness of the synthesized results.	6-7
**Reporting bias assessment**	14	Describe any methods used to assess risk of bias due to missing results in a synthesis (arising from reporting biases).	6
**Certainty assessment**	15	Describe any methods used to assess certainty (or confidence) in the body of evidence for an outcome.	6
**RESULTS**			
**Study selection**	16a	Describe the results of the search and selection process, from the number of records identified in the search to the number of studies included in the review, ideally using a flow diagram.	7
	16b	Cite studies that might appear to meet the inclusion criteria, but which were excluded, and explain why they were excluded.	8

Topic	No.	Item	Location where item is reported
**Study characteristics**	17	Cite each included study and present its characteristics.	8-9
**Risk of bias in studies**	18	Present assessments of risk of bias for each included study.	14
**Results of individual studies**	19	For all outcomes, present, for each study: (a) summary statistics for each group (where appropriate) and (b) an effect estimate and its precision (e.g. confidence/credible interval), ideally using structured tables or plots.	10-13
**Results of syntheses**	20a	For each synthesis, briefly summarise the characteristics and risk of bias among contributing studies.	14
	20b	Present results of all statistical syntheses conducted. If meta-analysis was done, present for each the summary estimate and its precision (e.g. confidence/credible interval) and measures of statistical heterogeneity. If comparing groups, describe the direction of the effect.	10-13
	20c	Present results of all investigations of possible causes of heterogeneity among study results.	15
	20d	Present results of all sensitivity analyses conducted to assess the robustness of the synthesized results.	0
**Reporting biases**	21	Present assessments of risk of bias due to missing results (arising from reporting biases) for each synthesis assessed.	14
**Certainty of evidence**	22	Present assessments of certainty (or confidence) in the body of evidence for each outcome assessed.	10-13
**DISCUSSION**			
**Discussion**	23a	Provide a general interpretation of the results in the context of other evidence.	14-15
	23b	Discuss any limitations of the evidence included in the review.	16
	23c	Discuss any limitations of the review processes used.	16
	23d	Discuss implications of the results for practice, policy, and future research.	16
**OTHER INFORMATION**			
**Registration and protocol**	24a	Provide registration information for the review, including register name and registration number, or state that the review was not registered.	5
	24b	Indicate where the review protocol can be accessed, or state that a protocol was not prepared.	5
	24c	Describe and explain any amendments to information provided at registration or in the protocol.	5
**Support**	25	Describe sources of financial or non-financial support for the review, and the role of the funders or sponsors in the review.	16
**Competing interests**	26	Declare any competing interests of review authors.	16
**Availability of data, code and other materials**	27	Report which of the following are publicly available and where they can be found: template data collection forms; data extracted from included studies; data used for all analyses; analytic code; any other materials used in the review.	16

S2. PRISMA 2020 Abstract Checklist

Topic	No.	Item	Reported?
**TITLE**			
**Title**	1	Identify the report as a systematic review.	Yes
**BACKGROUND**			
**Objectives**	2	Provide an explicit statement of the main objective(s) or question(s) the review addresses.	Yes
**METHODS**			
**Eligibility criteria**	3	Specify the inclusion and exclusion criteria for the review.	Yes
**Information sources**	4	Specify the information sources (e.g. databases, registers) used to identify studies and the date when each was last searched.	Yes
**Risk of bias**	5	Specify the methods used to assess risk of bias in the included studies.	No
**Synthesis of results**	6	Specify the methods used to present and synthesize results.	Yes
**RESULTS**			
**Included studies**	7	Give the total number of included studies and participants and summarise relevant characteristics of studies.	Yes
**Synthesis of results**	8	Present results for main outcomes, preferably indicating the number of included studies and participants for each. If meta-analysis was done, report the summary estimate and confidence/credible interval. If comparing groups, indicate the direction of the effect (i.e. which group is favoured).	Yes
**DISCUSSION**			
**Limitations of evidence**	9	Provide a brief summary of the limitations of the evidence included in the review (e.g. study risk of bias, inconsistency and imprecision).	No
**Interpretation**	10	Provide a general interpretation of the results and important implications.	Yes
**OTHER**			
**Funding**	11	Specify the primary source of funding for the review.	No
**Registration**	12	Provide the register name and registration number.	No

S3. Search keys and number of articles retrieved

Database	Search interface / Where to paste the query	Query String	Number of results
PubMed	Advanced Search Builder[1]<br>> paste query into the Query box	("glucose" OR "sugar") AND ("blood" OR "serum" OR "venous" OR "capillary" OR "glucometer" OR ("glucose" AND "kit") OR "glucose meter") AND ("saliva" OR "salivary" OR "glucose oxidase" OR "glucose-oxidase")	4175
Cochrane's Library	Advanced Search[2]<br>> Search tab<br>> Trials tab after running the search	("glucose" OR "sugar") AND ("blood" OR "serum" OR "venous" OR "capillary" OR "glucometer" OR ("glucose" AND "kit") OR "glucose meter") AND ("saliva" OR "salivary" OR "glucose oxidase" OR "glucose-oxidase")	554
Web of Science	Advanced Search[3]<br>> Fielded Search tab<br>> All Fields from the dropdown menu	("glucose" OR "sugar") AND ("blood" OR "serum" OR "venous" OR "capillary" OR "glucometer" OR ("glucose" AND "kit") OR "glucose meter") AND ("saliva" OR "salivary" OR "glucose oxidase" OR "glucose-oxidase")	4277
Embase	Advanced tab[4]	('glucose' OR 'sugar') AND ('blood' OR 'serum' OR 'venous' OR 'capillary' OR 'glucometer' OR ('glucose' AND 'kit') OR 'glucose meter') AND ('saliva' OR 'salivary' OR 'glucose oxidase' OR 'glucose-oxidase')	6710

[1] URL: https://pubmed.ncbi.nlm.nih.gov/advanced/
[2] URL: https://www.cochranelibrary.com/advanced-search
[3] URL: https://www.webofscience.com/wos/woscc/basic-search
[4] URL: https://www.embase.com/#advancedSearch/default

S4. Adapted QUADAS-2 signaling questions for paired salivary-blood glucose studies

Domain 1. Patient selection

1.1. Was a consecutive or random sample enrolled (avoids case-control spectrum bias)?
1.2. Were inclusion/exclusion criteria clearly defined? (e.g., fasting>=8 h); xerostomia/excessive gingival crevicular fluid excluded?
1.3. Were inappropriate exclusions avoided (e.g., excluding based on post-hoc salivary value)?

Judgement rule: Low risk if 1.1-1.3=Yes; High if any No; Unclear otherwise.

Domain 2. Indicator measurement (salivary glucose)

2.1. Was the saliva collection method standardized (unstimulated vs parotid; pre-rinse; seated; specified volume)?
2.2. Were pre-analytic conditions reported and appropriate (collection device, storage temperature and duration, time to assay)?
2.3. Was the assay method described (GOD-based), with calibration reported?
2.4. Were indicator results obtained without knowledge of the paired blood value (or was measurement automated), minimizing interpretive bias?

Judgement rule: Low if 2.1-2.3=Yes and 2.4=Yes/Unclear (since lab blinding may be implicit in automated assays); High if key pre-analytic factors are missing.

Domain 3. Reference measurement (blood glucose)

3.1. Was the blood sampling method specified (venous/capillary) and appropriate for fasting glucose?
3.2. Was the assay/device described, with calibration?
3.3. Were results obtained without knowledge of salivary values?

Judgement rule: As above.

Domain 4. Flow and timing (paired sampling)

4.1. Were saliva and blood drawn near-simultaneously after overnight fasting?
4.2. Was time between samples short and reported (e.g. <=15-30 min)?
4.3. Were all participants who provided one sample also providing the other (paired data completeness)?

Judgement rule: Low if 4.1-4.3=Yes; High if pairing or fasting timing is violated.

S5. Excluded articles

Stage	Number of excluded articles	Reason
Duplicate removal	6631	Duplicate
Title/Abstract Screening[1]	8984	Animal/non-human studies (n=1777)
		Neonatal/fetal/pregnancy-related studies (n=853)
		Biosensors/materials/devices (n=2096)
		Reviews/meta-analyses (n=557)
		Non-target biological/clinical studies (n=2471)
		Other sample types (n=90)
		Other oral topics (n=627)
		Wrong study type (n=617)
		Other miscellaneous topics (n=749)
Retrieval	24	No access to full text (even after directly contacting authors)
Eligibility assessment	4	Conference abstract
	32	Short fasting time
	16	No full-text version available

[1] The number of unique records excluded at the title/abstract screening stage is less than the sum of category counts, because individual records may meet multiple exclusion criteria. Category counts are therefore not mutually exclusive.

S6. Data on storing conditions, analysis methods, and volume of salivary samples

Study	Storing	Method	Volume
Gupta, et al. (2015)	preserved in an ice box	GOD-POD method	2 ml
Cui, et al. (2022)	pre-chilled polypropylene tubes on ice	GOD-POD (Glucose kit, Beijing Furui Runkang Biotechnology Co., Ltd., Beijing, China	no data
Wang, et al. (2017)	parotid saliva and mixed saliva were stored at −80◦C until use	GOD-POD, glucose test kit (Leadman Biochemistry Co., Ltd, Beijing, China	no data
Panda, et al. (2012)	sterile sample collection container, no data for cooling	GOD-POD method	no data
Forbat, et al. (1981)	plain tubes and stored at < 4°C	glucose oxidase method using a Beckmann glucose analyser	no data
Choundry, et al. (2022)	sterile, disposable plastic container, stored on ice	GOD-POD method in the Dirui-300B auto analyser	no data
Dhanya, et al. (2015)	sterile test tube, no data for cooling	glucose oxidase end point assay using Abcam's Glucose Assay Kit reagents.	no data
Ephraim, et al. (2019)	clean plastic saliva collection container	ELITech Diagnostic, GOD-POD method	no data
AlQusayer, et al. (2019)	collected non-invasiveliy, no data for cooling	NA	10 ml
Egboh, et al. (2022)	plastic vials	GOD end-point method	2 ml
Andersson-Borg, et al. (1998)	Lashley cups, stored on ice	enzymatic method (Cat. No 139041, Boerhinger Mannheim, Mannheim, Germany)	1,5 ml
Carramolino-Cuéllar, et al. (2017)	decanted, no data for cooling	glucose oxidase technique (Trinder GOD-POD Spinreact®, Gerona, Spain)	no data
Ganesan, et al. (2021)	cooled after centrifuged	GOD-POD method	3 ml
Harish, et al. (2018)	cooled after centrifuged	semi-automated Biochemistry analyzer using kits procured from ERBA diagnostics (Transasia Bio-Medicals Ltd, Germany)	no data
Harish, et al. (2019)	cooled after centrifuged	Biochemistry analyzer using the commercially available kit (Transasia BioMedicals Ltd, Germany). GOD-POD	no data
Kadashetti, et al. (2015)	sterile sample collection container, no data for cooling	GOD-POD method	no data
Manjrekar, et al. (2012)	no data for cooling	GOD POD method by using the Aggape Diagnostic kit	no data
Mrag, et al. (2020)	centrifuged, no data for cooling	AU680 (Beck- man-Coulter, USA)	no data
Mussavira, et al. (2015)	ice-chilled sterile polypropylene tube	Eco-Pak Glucose Kit (Accurex Biomedical)	no data
Nirmala, et al. (2019)	cooled after centrifuged	GOD-POD method	no data
Ravindran, et al. (2015)	ice carrier box	GLUCOSE-LS (Euro Diagnostic Systems Pvt. Ltd.) SA , GOD-POD method	1,5 ml
Shahbaz, et al. (2014)	sterile plastic container and stored over the ice	GOD end-point method	no data
Sharma, et al. (2020)	centrifuged, no data for cooling	Erba Chem-5 Plus v2 semi autoanalyzer	2 ml
Sharon, et al. (2020)	no data for cooling	GOD, colorimeter	no data

S7. Meta-regression for whole-mouth saliva and blood glucose samples

In the case where both groups (y-intercept: 11.249221820 (95% CI: 3.8521; 18.6464), slope: 0.002053316 (95% CI: -0.0001; 0.0042)) or only the diabetic group (y-intercept: 24.03240110 (95% CI: -11.3478; 59.4126), slope: -0.05827962 (95% CI: -0.2624; 0.1459)) were examined, the results were not significant, but the result was significant in the case of healthy patients (y-intercept: -101.979497 (95% CI: -210.2081; 6.2491), slope:1.260388 (95% CI: 0.0505; 2.4703)).

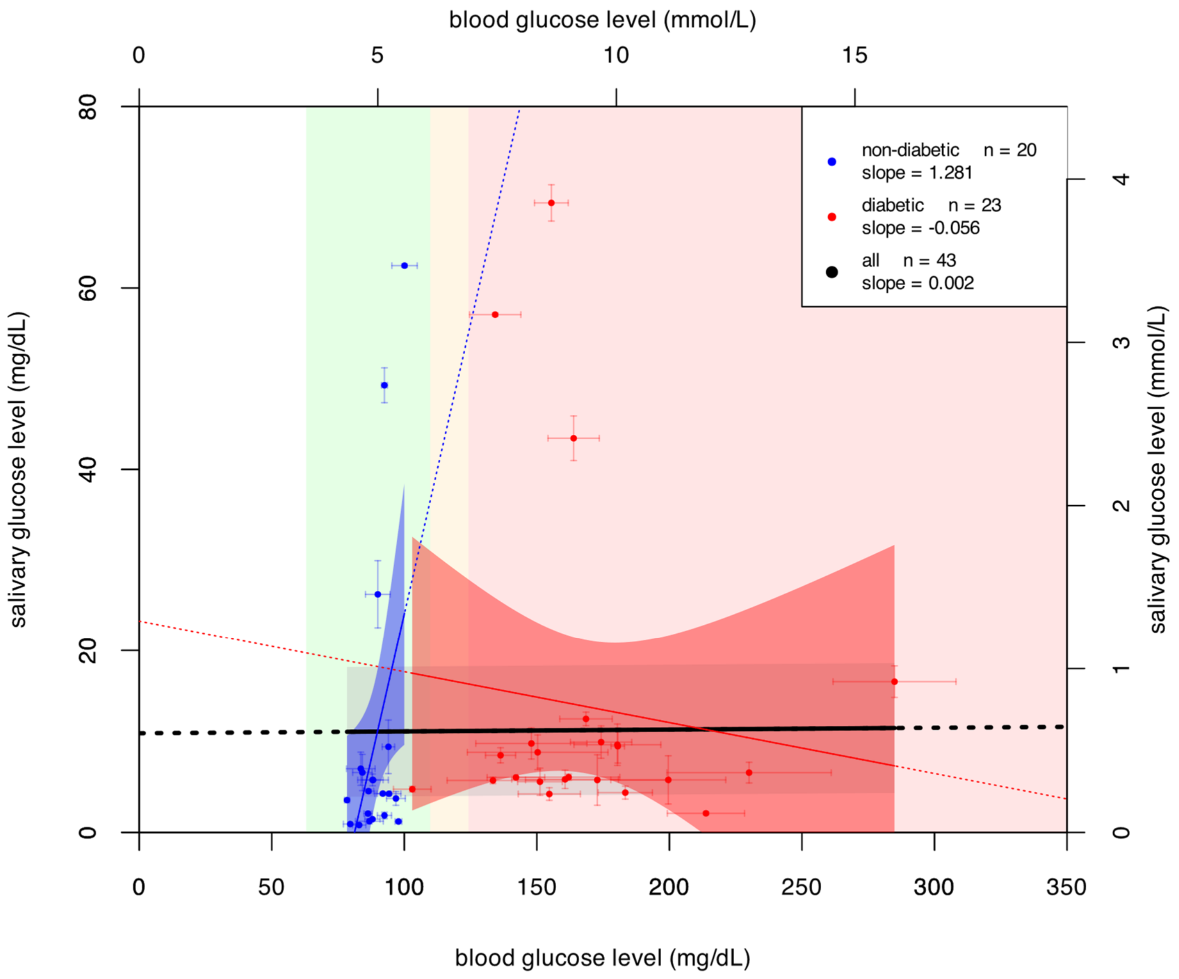

Supplement: Supplementary file 1 [file jcm-15-01829-s001.zip › jcm-4119592-supplementary.pdf]
